# TAZ inhibits osteoclastogenesis by attenuating TAK1/NF-κB signaling

**DOI:** 10.1038/s41413-021-00151-3

**Published:** 2021-07-12

**Authors:** Wanlei Yang, Xuanyuan Lu, Tan Zhang, Weiqi Han, Jianlei Li, Wei He, Yewei Jia, Kangxian Zhao, An Qin, Yu Qian

**Affiliations:** 1 Department of Orthopaedics, Shaoxing People’s Hospital (Shaoxing Hospital, Zhejiang University School of Medicine), Shaoxing, Zhejiang PR China; 2 Department of Orthopedic Surgery, Shanghai Key Laboratory of Orthopedic Implants, Shanghai Ninth People’s Hospital, Shanghai Jiao Tong University School of Medicine, Shanghai, China

**Keywords:** Bone, Bone quality and biomechanics

## Abstract

Osteoporosis is an osteolytic disorder commonly associated with excessive osteoclast formation. Transcriptional coactivator with PDZ-binding motif (TAZ) is a key downstream effector of the Hippo signaling pathway; it was suggested to be involved in the regulation of bone homeostasis. However, the exact role of TAZ in osteoclasts has not yet been established. In this study, we demonstrated that global knockout and osteoclast-specific knockout of TAZ led to a low-bone mass phenotype due to elevated osteoclast formation, which was further evidenced by in vitro osteoclast formation assays. Moreover, the overexpression of TAZ inhibited RANKL-induced osteoclast formation, whereas silencing of TAZ reduced it. Mechanistically, TAZ bound to TGF-activated kinase 1 (TAK1) and reciprocally inhibited NF-κB signaling, suppressing osteoclast differentiation. Collectively, our findings highlight an essential role of TAZ in the regulation of osteoclastogenesis in osteoporosis and its underlying mechanism.

## Introduction

Osteoporosis is a common osteolytic disease in the aging population and in postmenopausal women.^1^ Bone is a highly dynamic tissue that undergoes constant remodeling and is balanced via the processes of osteoclastic bone resorption and osteoblastic bone formation. An imbalance of these processes that favors excessive formation and/or activation of osteoclasts is the main cause of osteoporosis and other osteolytic conditions.^2^ Therefore, many studies have attempted to identify the molecules that regulate osteoclast formation and bone resorption.

Osteoclast formation is a stepwise process initiated by the binding of receptor activator of nuclear factor-κB ligand (RANKL) to its receptor RANK on monocytic/macrophage precursors. This results in the recruitment of the adapter proteins tumor necrosis factor receptor-associated factor 6 and TGF-β activated kinase 1 (TAK1) through ubiquitination or phosphorylation to form a signal complex. This complex further transmits signals and phosphorylates I-κBα, leading to the nuclear translocation of released nuclear factor-κB (NF-κB) subunits, such as p65, to the nucleus.^3^ Early signaling cascades culminate in the activation and upregulated expression of nuclear factor of activated T cells c1 (NFATc1), the master transcription factor that regulates the expression of key osteoclast genes involved in differentiation, precursor fusion and multinucleation, and the bone resorptive function of osteoclasts.^4^

Transcriptional coactivator with PDZ-binding motif (TAZ), also known as WW-domain containing transcription regulator-1, is a key downstream effector of the Hippo signaling pathway.^5^ In the canonical Hippo signaling pathway, TAZ localization and therefore function are regulated by the upstream kinases mammalian STE20-like protein kinase 1/2 (MST1/2) and large tumor suppressor homolog 1/2 (LATS1/2).^6^ TAZ is localized in both the cytoplasm and the nucleus of cells, and its distribution is governed by LATS1/2-dependent phosphorylation and binding to 14-3-3 proteins for subsequent degradation.^7,8^ Consistent with its dual localization, TAZ has been shown to exhibit localization-dependent functions. In the nucleus, TAZ regulates gene expression by interacting with members of the TEAD family of transcription factors.^9^ Conversely, cytoplasmic TAZ has been shown to bind Axin and Disheveled (DVL) to further promote β-catenin degradation by the proteasomal pathway,^10^ thereby antagonizing the β-catenin/Wnt signaling pathway.^11^ Reportedly, yes-associated protein (YAP), a paralog of TAZ, directly interacts with TAK1 and YAP/TAZ to attenuate NF-κB signaling by reducing the substrate accessibility of TAK1 in chondrocytes.^12^ Several studies have suggested the involvement of Hippo/TAZ signaling in bone homeostasis, particularly in the regulation of osteoblast formation and function.^13–16^ Our group has previously reviewed the emerging role of the Hippo signaling pathway in osteoclasts^17^ and found the gene expression of several members of the Hippo signaling pathway to be differentially regulated by RANKL during osteoclast formation. In particular, we observed a dramatic time-dependent decrease in the gene expression of TAZ (Supplementary Fig. 1) following RANKL stimulation, suggesting that TAZ may play an important role in the regulation of RANKL-induced osteoclast formation.

In this study, we showed for the first time that global and conditional deletion of TAZ induces low-bone mass phenotypes in mice owing to enhanced RANKL-induced osteoclast formation and bone resorption. Mechanistically, we identified TAK1 as an interacting partner for TAZ and established that the TAZ-TAK1 interaction is crucial for regulation of the NF-κB signaling pathway in osteoclasts.

## Results

### TAZ is downregulated in osteoporosis

**Fig. 1:**
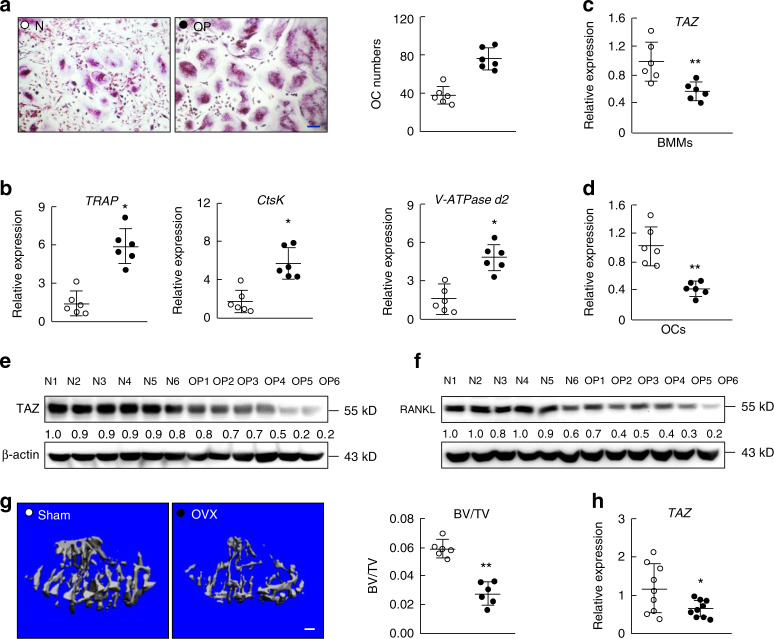
The expression of TAZ is downregulated in osteoporosis. **a** TRAP-staining and quantitation of multinucleated osteoclasts derived from BMMs from normal bone density (N, control group, underwent posterior internal fixation following thoracolumbar fracture) and osteoporotic (OP, underwent percutaneous kyphoplasty following thoracolumbar fracture) animals stimulated with 100 ng·mL^−1^ RANKL for 10 d. Scale bar denotes 10 μm. **b** Real-time qPCR analysis of the expression of osteoclast marker genes. Real-time qPCR analysis of the expression of the TAZ gene in BMMs (**c**) and osteoclasts (**d**) from the N and OP groups, respectively; *n* = 6. **e** and **f** Western blot analysis of the TAZ protein from BMMs and osteoclasts from the N and OP groups, respectively. **g** Three-dimensional micro-CT reconstructions of tibias from the sham (control) and ovariectomy (OVX) group. Scale bar denotes 200 µm. **h** Real-time qPCR analysis of the expression of the TAZ gene. In **a**−**f**, ○ and ● represent the N and OP groups, respectively. In **g** and **h**, ○ and ● represent the sham and OVX groups, respectively. An unpaired Student’s *t* test was performed. *P < 0.05, **P < 0.01. BMD, bone mineral density; BMM, bone marrow-derived macrophages

To determine the relationship between TAZ expression and osteoclasts in human osteoporosis, bone marrow specimens were collected from the thoracolumbar vertebrae in patients with thoracolumbar vertebral fractures with or without osteoporosis. Patient information is tabulated in Supplementary Table 1. In the OP group, bone marrow was harvested during percutaneous kyphoplasty procedures for osteoporotic vertebral compression fracture. In the control group, bone marrow was harvested during posterior internal fixation for thoracolumbar burst fracture in patients without osteoporosis. The bone marrow was cultured to obtain bone marrow-derived macrophages and osteoclasts. The results showed that osteoclast formation (Fig. 1a) and the expression of osteoclast marker genes (Fig. 1b) were higher in the OP group than in the normal bone density control group. Interestingly, the expression of the TAZ gene was downregulated in BMMs and osteoclasts from the OP group compared with those from the control group (Fig. 1c, d). In addition, the expression of the TAZ protein in BMMs and osteoclasts derived from the OP group was lower than that in BMMs and osteoclasts derived from the normal bone density control group (Fig. 1e, f). These results suggested that TAZ may play a role in osteolytic osteoporosis.

To further support the existence of a relationship between TAZ expression and human osteoporosis, osteoporotic (OP) and osteoarthritic (OA, control group) femoral head specimens obtained during surgery were examined. Patient information is tabulated in Supplementary Table 2. Three-dimensional (3D) reconstructions of micro-computed tomography (CT) scans and quantitative morphometric analyses of the OA and OP femoral heads were performed (Supplementary Fig. 2a). Histological and quantitative histomorphometric analyses (Supplementary Fig. 2b), as well as the determination of bone mineral density (BMD) by dual-energy X-ray absorptiometry (Supplementary Fig. 2c), further confirmed the lower bone mass trend in the OP femoral heads. Furthermore, we assessed the serum level of the bone resorption marker C-terminal telopeptide of type 1 collagen (CTX), which was reportedly higher in the OP group than in the OA group (Supplementary Fig. 2d). Consistent with the higher bone loss in the OP group, the expression of osteoclast marker genes, including *tartrate-resistant acid phosphatase (TRAP)*, *cathepsin K (CTSK)*, and *V-ATPase subunit d2* (*V-ATPase d2)*, was markedly upregulated in the OP group compared with the OA group (Supplementary Fig. 2f). The expression of osteogenic marker genes, including *runt-related transcription factor 2 (RUNX2)*, *alkaline phosphatase*, and *osteocalcin*, was significantly downregulated in the OP group compared with the OA group (Supplementary Fig. 2e). Interestingly, the expression of the *TAZ* gene was downregulated in the OP group compared with the OA group (Supplementary Fig. 2g), suggesting that TAZ may play a role in the pathogenesis of osteoporosis.

In addition, lower TAZ expression in osteoporosis was also evidenced by the OVX mouse model. The results showed a lower bone volume for tibias from OVX mice than for tibias from the sham control mice (Fig. 1g). Again, the expression of the TAZ gene was downregulated in the OVX group compared with the sham control group (Fig. 1h). Collectively, these results suggested that TAZ expression is downregulated in osteoporosis.

### Global gene knockout of TAZ induces an osteoporotic phenotype in mice

**Fig. 2:**
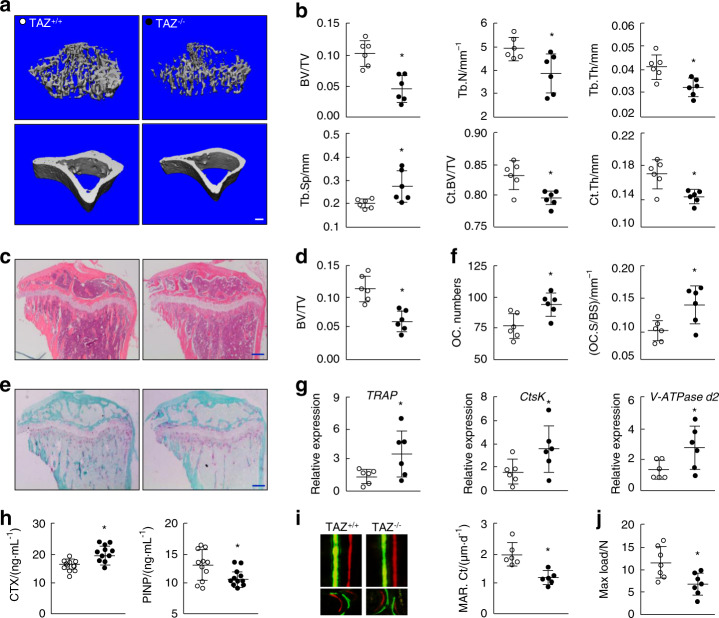
Mice subjected to TAZ global knockout (KO) exhibit an osteoporotic phenotype. **a** 3D micro-CT reconstructions of the trabecular region below the tibial plateau and the cortical region of WT (*TAZ*^*+/+*^) and KO (*TAZ*^*−/−*^) mice. Scale bar denotes 200 μm. **b** Quantitative morphometric assessment of micro-CT bone parameters, including trabecular BV/TV, Tb.N (mm^−1^), trabecular thickness (Tb.Th, mm), trabecular separation (Tb.Sp, mm), cortical region BV/TV (Ct. BV/TV) and cortical thickness (Ct. Th, mm). **c** Histological H&E staining of tibial sections from *TAZ*^*+/+*^ and *TAZ*^*−/−*^ mice. Scale bar 200 µm. **d** Quantitative histomorphometric assessment of trabecular BV/TV based on H&E-stained tibial sections. **e** Histological TRAP staining of tibial sections from *TAZ*^*+/+*^ and *TAZ*^*−/−*^ mice. Scale bar denotes 200 μm. **f** Quantitative assessment of osteoclast numbers (OC.N/BS) and osteoclast surface relative to total bone surface (OC.S/BS) based on TRAP-stained tibial sections. **g** Real-time qPCR analysis of the expression of osteoclast marker genes in forelimb bones. **h** ELISA assessment of serum CTX and P1NP levels in *TAZ*^*+/+*^ and *TAZ*^*−/−*^ mice. **i** Periosteal mineral apposition rate (MAR) by calcein-alizarin red double labeling. The upper panels represent cortical bone (Ct), and the lower panels represent trabecular bone (Tb). **j** Mechanical three-point bending tests of maximum load bearing of the tibial bone. ○ and ● represent the *TAZ*^*+/+*^ and *TAZ*^*−/−*^ groups, respectively. An unpaired Student’s *t*-test was performed. *P < 0.05

To determine the potential role of TAZ in osteoporosis, knockout mice with global deletion of the *TAZ* gene were generated (breeding information is presented in Supplementary Table 3, genotyping is presented in Supplementary Fig. 3a, and KO efficiency is presented in Supplementary Fig. 3b). *TAZ*^*−/−*^ (KO) mice were smaller in size (Supplementary Fig. 3c) and correspondingly lighter (Supplement Fig. 3i) than *TAZ*^*+/+*^ (WT) control mice. Polycystic kidney disease and emphysema were also observed in *TAZ*^*−/−*^ mice (Supplementary Fig. 3d), consistent with previous reports.^18,19^ Morphological micro-CT assessment was then carried out to assess the bone phenotype. Compared to *TAZ*^*+/+*^ mice, *TAZ*^*−/−*^ mice exhibited lower trabecular bone density and thinner cortical bone in the tibia based on reconstructed 3D micro-CT images (Fig. 2a). Assessment of quantitative morphometric bone parameters revealed significant reductions in BV/TV, Tb.N, Tb.Th, Ct. BV/TV, and Ct. Th and marked increases in Tb.Sp in *TAZ*^*−/−*^ mice; these bone changes were consistent with the osteoporotic phenotype (Fig. 2b). In fact, BV/TV was reduced from 0.109 4 ± 0.021 0 in *TAZ*^*+/+*^ mice to 0.056 2 ± 0.017 1 in *TAZ*^*−/−*^ mice (*P* < 0.05). 3D micro-CT reconstruction images and quantitative morphometric assessments of the vertebral bone (Supplementary Fig. 3e, f) and the skull (Supplementary Fig. 3g, h) further supported the osteoporotic phenotype in *TAZ*^*−/−*^ mice. The bone tissue microarchitecture was further assessed by histological staining of tissue sections with H&E. The low-bone mass osteoporotic phenotype of *TAZ*^*−/−*^ mice was evident in H&E-stained tissue samples (Fig. 2c) and confirmed by quantitative histomorphometric analysis of BV/TV (Fig. 2d). Together, these results demonstrated that global knockout of the *TAZ* gene in mice results in an osteoporotic phenotype.

Elevated osteoclast formation and bone resorption are often the underlying causes of low-bone mass osteoporotic phenotypes. Thus, tibial bone sections were stained with TRAP to assess osteoclast numbers and activity. As shown in Fig. 2e, f, tibial sections from *TAZ*^*−/−*^ mice exhibited elevated numbers of osteoclasts and greater osteoclastic bone resorptive activity than *TAZ*^*+/+*^ mice. This observation was in line with elevated gene expression profiles of osteoclast marker genes (Fig. 2g). In addition, serum levels of CTX (Fig. 2h) and TRAP (Supplementary Fig. 3), markers of bone resorption, were also elevated in *TAZ*^*−/−*^ mice. Previous reports have demonstrated a key role for TAZ in osteogenesis.^15,16^ Thus, we also assessed the effect of *TAZ*^*−/−*^ on osteoblast-mediated bone formation. Dynamic bone histomorphometric analysis following calcein green and alizarin red double labeling indicated that the bone mineral apposition rate (MAR) of both cortical and trabecular bone was decreased (Fig. 2i and Supplementary Fig. 3k), with reduced serum levels of procollagen type 1N propeptide (P1NP), a marker of bone formation, in *TAZ*^*−/−*^ mice (Fig. 2h). Finally, a biomechanical three-point bending test showed that the maximum load (Fig. 2j) and stiffness (Supplementary Fig. 3l) of the tibia in *TAZ*^*−/−*^ mice were significantly lower than those of the tibia in *TAZ*^+/+^ mice, suggesting that the bones from *TAZ*^*−/−*^ mice may fracture more easily. Collectively, these results suggest that under the normal physiological bone remodeling process, TAZ may exert a suppressive effect on osteoclast formation and bone resorption and a stimulatory effect on osteoblast-mediated bone formation. The loss of TAZ in global KO mice induces an osteoporotic phenotype due to elevated osteoclast numbers and excessive bone resorption, with a concomitant decrease in osteoblast-mediated bone formation. However, global TAZ deletion leads to runting and several nonskeletal pathologies (PKD and others), making it difficult to determine the direct effects of TAZ deletion in bone relative to secondary effects on other organs. Therefore, we further generated TAZ osteoclast-specific conditional KO mice.

### Osteoclast-specific conditional KO of TAZ in mice results in an osteoporotic phenotype

**Fig. 3:**
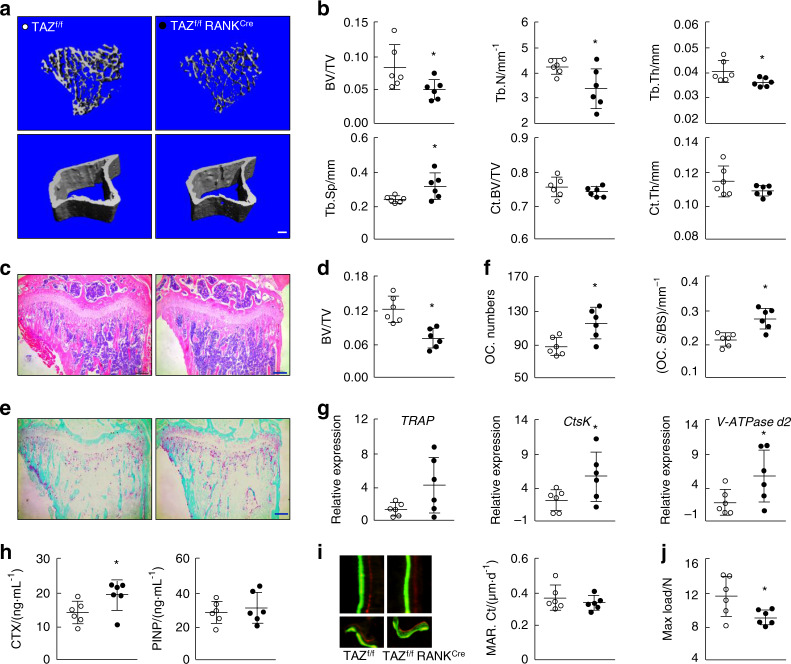
Osteoclast-specific (RANKCre) conditional knockout of TAZ results in a low-bone mass osteoporotic phenotype. **a** 3D micro-CT reconstructions of the trabecular region below the tibial plateau and the cortical region of *TAZ*^*f/f*^ (WT) and *TAZ*^*f/f*^*RANK*^*Cre*^ (cKO) mice. Scale bar denotes 200 μm. **b** Quantitative morphometric assessment of micro-CT bone parameters, including trabecular BV/TV, Tb.N (mm^−1^), Tb.Th (mm), Tb.Sp (mm), Ct. BV/TV and Ct. Th (mm). **c** Histological H&E staining of tibial sections. Scale bar denotes 200 μm. **d** Quantitative histomorphometric assessment of trabecular BV/TV based on H&E-stained tibial sections. **e** Histological TRAP staining of tibial sections. Scale bar denotes 200 μm. **f** Quantitative assessment of trabecular OC.N/BS and OC.S/BS based on TRAP-stained tibial sections. **g** Real-time qPCR analysis of the expression of osteoclast marker genes using RNA extracted from forelimb bones. **h** ELISA assessment of serum CTX and P1NP levels. **i** MAR determined by calcein-alizarin red double labeling. **j** Mechanical three-point bending tests of maximum load bearing of the tibial bone. ○ and ● represent the *TAZ*^*f/f*^ and *TAZ*^*f/f*^*RANK*^*Cre*^ groups, respectively. *n* = 6 for each group. An unpaired Student’s *t* test was performed. *P < 0.05

To further clarify the role of TAZ in osteoclasts, mice with conditional KO (cKO) of *TAZ* specifically in osteoclast lineage cells were generated using RANK^Cre^ transgenic mice, as illustrated in Supplementary Fig. 4a. Genotype and KO efficiency data are provided in Supplementary Fig. 4b, c, respectively. Similar to global *TAZ*^*−/−*^ mice, *TAZ*^*f/f*^*RANK*^*Cre*^ (cKO) mice showed thinly distributed trabecular bone in the tibia but no observable difference in cortical bone thickness (Fig. 3a) when compared with *TAZ*^*f/f*^ (negative for Cre recombinase) WT mice. Morphometric analyses of trabecular and cortical bone parameters confirmed the reduced bone mass in trabecular bone but not cortical bone (Fig. 3b).

Again, histological and histomorphometric assessment of H&E- and TRAP-stained tibial bone sections further verified the low-bone mass phenotype observed in the *TAZ*^*f/f*^*RANK*^*Cre*^ mice (Fig. 3c, d) as a result of elevated osteoclast numbers and excessive osteoclastic bone resorption (Fig. 3e, f and Supplementary Fig. 4d). Gene expression profiling of osteoclast marker genes in the foreleg bone by real-time PCR showed upregulated expression of *TRAP*, *CtsK*, and *V-ATPase d2* in *TAZ*^*f/f*^*RANK*^*Cre*^ mice (Fig. 3g). The elevated serum levels of CTX in *TAZ*^*f/f*^*RANK*^*Cre*^ mice compared to *TAZ*^*f/f*^ mice further confirmed their higher bone resorption activity (Fig. 3h). No difference in serum P1NP content was found between *TAZ*^*f/f*^ and *TAZ*^*f/f*^*RANK*^*Cre*^ mice. Dynamic bone histomorphometric analysis following calcein green and alizarin red double labeling indicated that the MARs of both cortical and trabecular bone were not different between *TAZ*^*f/f*^ and *TAZ*^*f/f*^*RANK*^*Cre*^ mice (Fig. 3i and Supplementary Fig. 4e). The three-point bending test further demonstrated significantly reduced tibial stiffness in *TAZ*^*f/f*^*RANK*^*Cre*^ mice, but no difference was found in the maximum load bearing parameter (Fig. 3j and Supplementary Fig. 4f). Together, these results indicated that the specific deletion of TAZ in osteoclasts results in a low-bone mass phenotype reminiscent of osteoporosis due to elevated numbers and activation of osteoclasts.

### TAZ suppressed RANKL-induced osteoclastogenesis in vitro

**Fig. 4:**
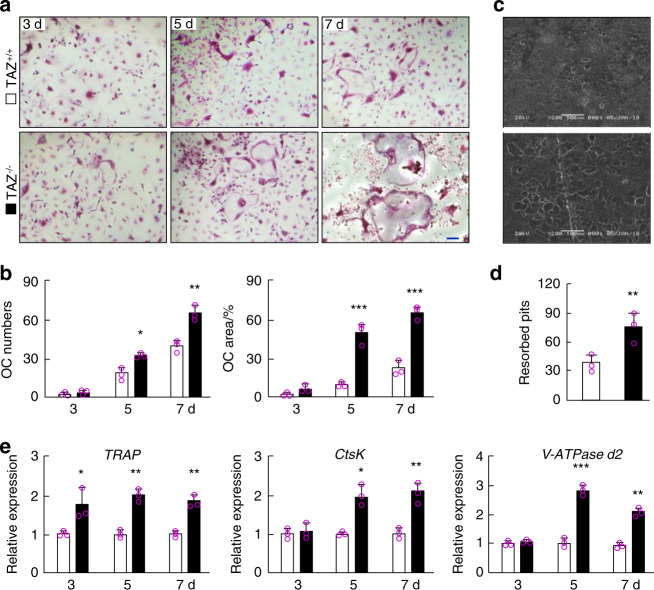
TAZ gene knockout enhances osteoclast differentiation and bone resorption. **a** TRAP-stained multinucleated osteoclasts derived from *TAZ*^*+/+*^ and *TAZ*^*−/−*^ BMMs stimulated with 100 ng·mL^−1^ RANKL for 3, 5, and 7 d. Scale bar 10 μm. **b** Quantitative measurement of the number of TRAP-positive multinucleated osteoclasts with three or more nuclei and their average size (area) as a percentage of the total well area. **c** Scanning electron micrographs of bone resorption pits. Scale bar denotes 100 μm. **d** Quantitative measurement of the number of resorption pits. **e** Real-time qPCR analysis of the expression of osteoclast marker genes. □ and ■ represent the *TAZ*^*+/+*^ and *TAZ*^*−/−*^ groups, respectively. One-way (**d**) or two-way (**b** and **e**) ANOVA followed by a post hoc Tukey test was performed. *P < 0.05, **P < 0.01, and ***P < 0.001

To further understand the cellular effects of TAZ on osteoclast differentiation and bone resorption, in vitro cellular and biochemical analyses were carried out. We first examined the differentiation potential of osteoclast precursor cells isolated from *TAZ*^*+/+*^ and *TAZ*^*−/−*^ mice. BMMs were stimulated with RANKL for 7 d, and their differentiation into mature osteoclasts was assessed by TRAP staining. As shown in Fig. 4a, b, *TAZ*^*−/−*^ BMMs formed more TRAP-positive multinucleated osteoclasts at days 5 and 7 than BMMs derived from *TAZ*^*+/+*^ littermates, suggesting higher precursor cell fusion and/or cell spreading capacity.

We thus examined whether *TAZ*^*−/−*^ osteoclasts exhibited alterations in bone resorption capacity. When the same numbers of BMMs were seeded in bone disks, as shown in SEM micrographs and the subsequent quantitative analysis, *TAZ*^*−/−*^ BMMs showed elevated bone resorptive function, leaving behind more resorption pits and trails on the bone disks than *TAZ*^*+/+*^ BMMs (Fig. 4c, d). To further exclude the effect of osteoclast formation on bone resorption, we activated both *TAZ*^*+/+*^ and *TAZ*^*−/−*^ BMMs with RANKL until osteoclast formation, and then equal numbers of mature *TAZ*^*+/+*^ and *TAZ*^*−/−*^ osteoclasts were seeded on bone disks (Supplementary Fig. 5). Interestingly, the bone resorption pits showed no difference between these two types of osteoclasts, which indicated that the loss of TAZ affected osteoclast formation but had no effect on bone resorption activity. The increased number of resorption pits maybe not due to increased bone resorption activity but rather due to elevated osteoclast numbers. Real-time PCR analysis of osteoclast marker gene expression revealed upregulation of *TRAP* expression at all time points (days 3, 5 and 7) and upregulation of *CtsK* and *V-ATPase d2* in the later stages of osteoclast differentiation (i.e., days 5 and 7), during which precursor cells were undergoing cellular fusion, in the *TAZ*^*−/−*^ group when compared with the *TAZ*^*+/+*^ group (Fig. 4e). These effects of the loss of TAZ on osteoclast formation, gene expression and bone resorption were recapitulated when using BMMs derived from *TAZ*^*f/f*^*RANK*^*Cre*^ mice (Supplementary Fig. 6).

To further confirm the cellular effects of TAZ on RANKL-induced osteoclast formation and activity, BMMs derived from WT mice were transduced with lentiviral particles that led to overexpression or silencing of the *TAZ* gene. *TAZ* overexpression suppressed RANKL-induced osteoclastogenesis (Supplementary Fig. 7a) and downregulated osteoclast marker gene expression (Supplementary Fig. 7b). Consistent with the inhibitory effect on osteoclast formation, bone resorption was similarly reduced following the overexpression of TAZ (Supplementary Fig. 7c). TAZ gene knockdown (silencing efficiency in Supplementary Fig. 8a, b) by silencing resulted in effects similar to those observed with KO BMMs, demonstrating enhanced RANKL-induced osteoclast formation and osteoclast marker gene expression (Supplementary Fig. 8c–e). The results from the overexpression and silencing experiments provided further evidence that TAZ inhibits RANKL-induced osteoclast differentiation. Collectively, these results confirmed that TAZ plays a physiological inhibitory role against RANKL-induced osteoclast formation.

### TAZ interacts with TAK1 during RANKL-induced osteoclast differentiation

**Fig. 5:**
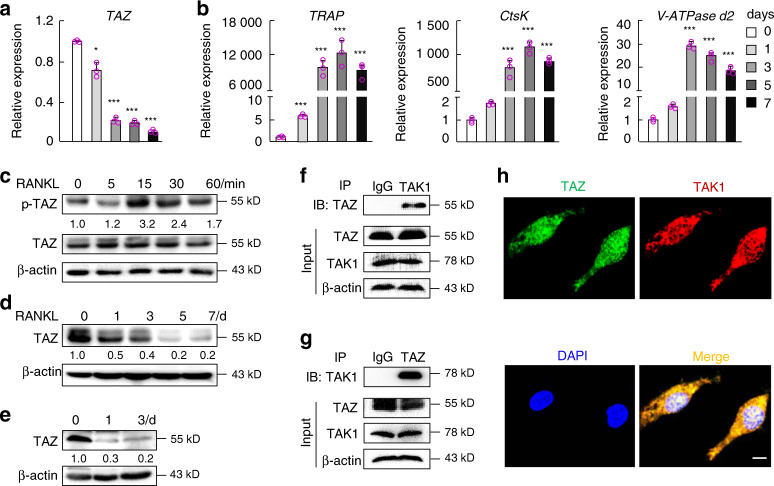
TAZ interacts with TAK1. Real-time qPCR analysis of the expression of (**a**) TAZ and (**b**) osteoclast marker genes in BMMs stimulated with RANKL for 1, 3, 5, and 7 d. Unstimulated BMMs were used as a mock control. **c** Western blot analysis of the phosphorylation status of TAZ in BMMs following stimulation with RANKL for 5, 15, 30, and 60 min. Unstimulated BMMs were used as a mock basal control. **d** Protein expression of TAZ in BMMs stimulated with RANKL for 1, 3, 5, and 7 d and **e** RAW264.7 cells stimulated with RANKL for 1 and 3 d. Unstimulated cells were used as a mock control. **f**, **g** Coimmunoprecipitation analysis of TAZ and TAK1 protein interactions. **h** Colocalization between TAZ and TAK1 in BMMs by confocal microscopy. Scale bar denotes 5 μm. *n* = 3. One-way ANOVA was followed by a post hoc Tukey test. *P < 0.05, **P < 0.01, and ***P < 0.001. BMMs, bone marrow-derived macrophages

After establishing the cellular effects of TAZ, we aimed to elucidate the underlying molecular mechanism. First, we examined the gene expression profile of *TAZ* during RANKL-induced osteoclast differentiation. As shown in Fig. 5a, b, *TAZ* expression was downregulated in a time-dependent manner, a trend that was opposite to that observed for osteoclast marker genes, whose expression was upregulated in response to RANKL stimulation (Fig. 5a, b). By using total cellular proteins extracted from BMMs stimulated with RANKL for up to 60 min, we observed the time-dependent phosphorylation of TAZ (Fig. 5c). Furthermore, prolonged stimulation with RANKL (i.e., during the process of osteoclast formation) resulted in decreased expression of the TAZ protein (indicative of protein degradation) in both stimulated BMMs and RAW264.7 cells (Fig. 5d, e respectively). Thus, these findings suggest that RANKL stimulation may induce the phosphorylation of TAZ and lead to the targeted degradation of TAZ during osteoclast differentiation.

Next, we aimed to identify cofactors/effectors that interact with TAZ in osteoclasts. A previous study demonstrated that reciprocal suppression occurs between YAP/TAZ and the NF-κB pathway, regulating cartilage degradation during the pathogenesis of osteoarthritis. Thus, YAP directly binds to TAK1 and attenuates the NF-κB signaling pathway.^12^ Based on these findings, we hypothesized that TAZ could bind to TAK1 and further inhibit the NF-κB pathway in osteoclasts. The endogenous interaction between TAZ and TAK1 was further confirmed using co-IP assays (Fig. 5f, g). Furthermore, confocal data demonstrated the colocalization of TAZ and TAK1 (Fig. 5h and Supplementary Fig. 9).

### Reciprocal suppression between TAZ and the NF-κB pathway regulated osteoclast differentiation

**Fig. 6:**
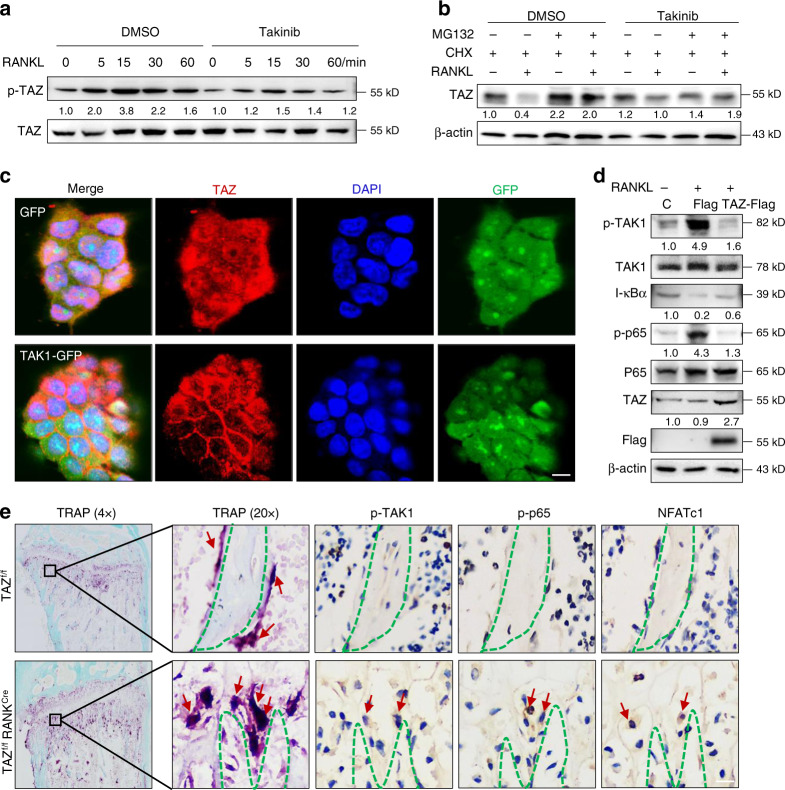
Reciprocal suppression between TAZ and NF-κB pathway components regulates osteoclastogenesis. **a** Western blot analysis evaluating the effect of takinib on TAZ phosphorylation during RANKL stimulation in BMMs. **b** Western blot analysis evaluating the effect of takinib on TAZ degradation with CHX pretreatment for 2 h followed by treatment with RANKL for 6 h along with MG132 in BMMs. **c** TAZ immunofluorescence staining of HEK 293T cells transfected with the GFP empty vector or TAK1-GFP overexpression plasmid. Scale bar denotes 10 μm. **d** Western blot analysis evaluating the effect of TAZ overexpression on p-TAK1, I-κBα, and p-p65 levels upon RANKL stimulation for 15 min in BMMs. **e** TRAP staining and p-TAK1, p-p65, and NFATc1 immunofluorescence staining of cancellous bone from the proximal tibia of TAZ^f/f^ and TAZ^f/f^RANK^cre^ mice. Scale bar denotes 20 μm. BMMs, bone marrow-derived macrophages; CHX, cycloheximide

To determine the relevance of the TAZ-TAK1 interaction, we first examined the effect of TAK1 inhibition on the TAZ protein using the specific inhibitor takinib via western blot analysis. In the BMMs, TAZ was phosphorylated by RANKL upon short-duration stimulation, and this effect was attenuated by takinib (Fig. 6a). Phosphorylated TAZ remained in the cytoplasm, interacting with 14-3-3 proteins to further mediate functions or degradation. Hence, attenuation of TAZ phosphorylation by takinib may result in the accumulation of the TAZ protein.^8^ Next, we evaluated the effect of takinib on TAZ degradation upon cycloheximide (CHX) pretreatment for 2 h, followed by treatment with RANKL for 6 h along with MG132. The results showed that RANKL stimulation reduced TAZ protein expression, and this reduction was rescued by takinib (Fig. 6b). Furthermore, the reduced expression of the TAZ protein decreased upon increased RANKL stimulation for 48 h (Supplementary Fig. 10a). In HEK 293T cells, inhibition of TAK1 with takinib resulted in the translocation of TAZ into the nucleus (Supplementary Fig. 10b), and these results were further confirmed by the nucleocytoplasmic separation data (Supplementary Fig. 10c). Conversely, compared with the cells transfected with the GFP empty vector, the cells transfected with the TAK1-GFP vector predominantly showed TAZ localization to the cytoplasm (Fig. 6c). In addition, silencing TAK1 led to suppression of TAZ phosphorylation induced by RANKL (Supplementary Fig. 10d). The decline in TAZ protein levels stimulated by RANKL for 48 h was also attenuated by TAK1 silencing (Supplementary Fig. 10e). These findings reveal that TAK1 binds to TAZ and inhibits TAZ activity.

We further explored the effect of TAZ on the TAK1/NF-κB pathway. RANKL phosphorylates TAK1 and degrades I-κBα to release p65 into the nucleus. During RANKL stimulation, the activated TAK1/NF-κB signaling pathway was suppressed by the overexpression of TAZ via a lentivirus (Fig. 6d). Inhibition of TAZ with verteporfin (VP) upregulated the activity of NF-κB luciferase (Supplementary Fig. 10f). These results were corroborated by demonstrating that the overexpression or knockout of TAZ reduced or upregulated the mRNA and protein expression of NFATc1, respectively, during RANKL-induced osteoclastic differentiation (Supplementary Fig. 10g, h). More importantly, to visualize osteoclasts in bone sections, we performed TRAP and immunohistochemical staining of serial sections. TRAP-positive osteoclasts were observed on the surface of trabecular bone; p-TAK1-, p-p65-, and NFATc1-positive staining was also observed at the same position. Osteoclast-conditional knockout of TAZ promoted the phosphorylation of TAK1 and p65, as well as the expression of NFATc1 (Fig. 6e), as determined by immunohistochemistry, indicating that knockout of TAZ also activated the TAK1/NF-κB signaling pathway in vivo. Collectively, these data confirmed that the interaction between TAZ and TAK1 reciprocally suppresses TAZ and the TAK1/NF-κB pathway, regulating osteoclast differentiation.

## Discussion

In this study, we identified TAZ as a novel regulator of bone homeostasis that acts via the suppression of RANKL-induced osteoclast formation. The global deletion and osteoclast-specific deletion (RANK^Cre^) of TAZ resulted in an osteoporotic low-bone mass phenotype owing to elevated osteoclast formation and bone resorption in vivo and in vitro. Additionally, biochemical analyses identified TAK1 as an interaction partner for TAZ, suggesting a model whereby the interaction of TAZ-TAK1 reciprocally suppresses the interaction between TAZ and the TAK1/NF-κB pathway to regulate osteoclast differentiation.

TAZ is a downstream effector of the Hippo signaling pathway and was identified as an interacting partner for cytoplasmic 14-3-3 proteins that shares sequence homology with YAP, another downstream Hippo effector.^8,20^ Further studies showed that TAZ exhibits transcriptional regulatory activity, interacting with a number of transcription factors to regulate the expression of genes involved in embryogenesis, development of muscle, fat, lung, heart, limbs and bone, and mesenchymal stem cell differentiation.^16,21–24^ In terms of bone development and homeostasis, zebrafish with TAZ deletion lack ossification and ventral curvature.^16,18^ Numerous in vitro studies have shown a role for TAZ in promoting osteoblast differentiation and function.^25–27^ Targeted overexpression of TAZ in osteoblasts increased bone mass in vivo,^28^ whereas *TAZ* cKO in osteoblasts (*Osterix*^*Cre*^) resulted in an osteoporotic phenotype, and osteoblast-specific *TAZ/YAP* double cKO led to neonatal fatality due to debilitating skeletal fragility and deformity that mimicked osteogenesis imperfecta.^15^ In both models, there were increased osteoclast numbers. In another study, deletion of *YAP* and *TAZ* in mature osteoblasts and osteocytes using *Dmp1*^*Cre*^ transgenic mice also yielded a low-bone mass phenotype due to reductions in osteoblast formation and bone formation activity and an increase in osteoclast numbers.^14^ However, in another study, compound heterozygous *TAZ*^*+/−*^ and *PKD1*^*+/−*^ (polycystin 1) mice exhibited an additive bone loss effect due to a reduction in osteoblast-mediated bone formation.^29^ Mice with deletion of PGC-1α in skeletal stem cells (SSCs) exhibited reduced bone mass as a result of lower osteoblast numbers and function. The study showed that the loss of PGC-1α in SSCs significantly inhibited the induction of TAZ during osteogenic differentiation.^30^ Interestingly, mice with global knockout of *TAZ* showed only partial lethality, and the mice that survived exhibited a slightly runted skeleton and only minor skeletal anomalies.^18,19,31^

In contrast, our *TAZ*^*−/−*^ global KO and osteoclast-specific (RANK^Cre^)*TAZ* cKO mice displayed osteoporotic low-bone mass phenotypes, which is in line with other reports. Furthermore, to our knowledge, our study is the first to report that the osteoporotic phenotype caused by the loss of TAZ is the result of enhanced osteoclast formation and bone resorption in vivo. However, we examined only tibia micro-CT at the 8-week time point. The thickness of the cortical bone could be further assessed at 12 weeks or later. The thickness of the cortical bone in TAZ^f/f^ RANK^cre^ might decrease upon an increase in osteoclast formation at 12 weeks or later. RANK is expressed in multiple cell types in addition to osteoclasts, which cannot completely exclude the effect of TAZ deletion in other cell types on bone volume. Therefore, CtsK^cre^ needs to be assessed in the future. In vitro, the cellular effect of loss of TAZ on osteoclast formation and bone resorption was more apparent. By using BMMs derived from *TAZ*^*−/−*^ mice, we observed elevated osteoclast formation, and the resulting osteoclasts derived from *TAZ*^*−/−*^ BMMs were significantly larger than their WT counterparts, suggesting enhanced/elevated fusion capacity of precursor cells. By using *TAZ* overexpression and silencing approaches, we further confirmed these effects, providing evidence that TAZ may act as a physiological regulator to protect against excessive RANKL-induced osteoclast formation. Based on its significant role in maintaining bone development, promoting osteoblast formation, and suppressing osteoclast formation, TAZ, as a protective molecule, plays a critical role in the maintenance of bone homeostasis.

Notably, the modulation of NF-κB signaling by RANKL is a critical mechanism controlling osteoclast-mediated bone homeostasis. A previous study revealed reciprocal suppression between YAP/TAZ and the NF-κB signaling pathway in the regulation of osteoarthritic chondrocyte degradation.^12^ However, the regulatory interaction between TAZ and the NF-κB signaling pathway in osteoclasts remains unknown. The mutual modulation of TAK1 and TAZ is still controversial. TAK1 was reported to bind to and stabilize YAP/TAZ and thus promote their nuclear translocation in bone marrow mesenchymal stem cells and cancer cells.^32,33^ Here, our data reveal reciprocal suppression between TAZ and TAK1 in osteoclasts. RANKL can phosphorylate both TAZ and TAK1. The inhibition or silencing of TAK1 with takinib attenuated TAZ phosphorylation, whereas TAZ overexpression also attenuated the TAK1 phosphorylation induced by RANKL. Previous studies reported the two main regulatory pathways of TAZ. As a transcriptional coactivator located in the nucleus, TAZ binds to transcription factors such as RUNX2 to promote osteoblast differentiation.^16^ Second, as a signaling molecule present in the cytoplasm, TAZ is involved in the transmission of phosphorylation signals.^34^ In the cytoplasm, the interaction between TAZ and DVL prevents the CK1δ/ɛ (casein kinase)-mediated phosphorylation of DVL, thereby inhibiting Wnt/β-Catenin signaling.^11^ The current study demonstrated that the reciprocal suppression between TAZ and NF-κB signaling, which occurs via phosphorylation-induced signal transmission between TAZ and TAK1 during RANKL stimulation, induced osteoclast differentiation. Our finding that TAZ regulates the activity of the NF-κB signaling pathway may provide additional insights into the manner in which the NF-κB signaling pathway could be fine-tuned and precisely controlled during osteoclast-mediated osteoporosis.

Moreover, TAZ phosphorylation is dependent on activation of the Hippo signaling cascade. At the core of Hippo signaling is a kinase cascade in which the serine/threonine kinase 20-like proteins MST1/2 phosphorylate and activate LATS1/2 kinase proteins. Subsequently, the LATS1/2 kinases phosphorylate and inactivate the effector protein TAZ (Ser89), enhancing their interaction with 14-3-3 proteins as well as their degradation.^8,35^ In our study, we observed that RANKL stimulation induced rapid phosphorylation of TAZ. Blocking protein synthesis using CHX resulted in a decrease in TAZ protein levels during RANKL stimulation for 6 h; however, the decreased TAZ protein levels were reversed by inhibiting protein degradation using MG132. Furthermore, sustained stimulation with RANKL during osteoclast formation resulted in a time-dependent decrease in the expression of the TAZ protein, suggesting that TAZ undergoes degradation. TAZ degradation is dependent on sequential phosphorylation at Ser311 by LATS1/2, followed by phosphorylation at Ser314 by CK1. This combinatorial phosphorylation recruits the SCFβ-TrCP E3 ligase that polyubiquitinates TAZ, thus targeting it for proteasomal degradation.^18,36^ However, whether this sequence of events and the activation of the Hippo signaling pathway under RANKL stimulation occur in osteoclasts warrants further investigation. Furthermore, in this study, we showed that RANKL triggers TAZ degradation through TAK1. Both TAZ and TAK1 have been implicated in responses to mechanical stimuli.^27,37^ Loss of mechanical load is a pivotal mechanism of osteoclastic activation and disuse osteoporosis.^38,39^ This suggests that TAZ may also play a significant role in disuse osteoporosis. In addition, our results also showed a decrease in TAZ mRNA levels during osteoclast formation and osteoporosis. These results, which may show regulation of TAZ transcription during osteoclast differentiation, warrant further investigation.

In conclusion, we uncovered that TAZ plays a novel role in the regulation of RANKL-induced osteoclast formation, as well as the pathogenesis of osteoporosis, by antagonizing the NF-κB signaling pathway. We identified TAK1 as an interacting partner for TAZ and demonstrated that this interaction reciprocally suppresses TAZ and TAK1 signaling to inhibit osteoclast differentiation. The role of proteasome-mediated posttranslational regulation between TAZ and NF-κB in the control of osteoclast differentiation is essential, in addition to transcriptional regulation. Collectively, our results provide evidence that TAZ is a critical regulator of bone homeostasis and suggest that TAZ is a potential therapeutic target for the treatment of osteoporosis.

## Materials and methods

### Study approval

Human studies were approved by the Ethics Committee of Shaoxing People’s Hospital (NO. 2017009 and NO. 2019KY15901). All participating subjects signed informed consent. All in vivo animal experimentations and protocols were approved by the Zhejiang University Institutional Animal Care and Use Committee (NO. ZJU20170370).

### Generation of TAZ global knockout (KO) mice and osteoclast-specific TAZ conditional knockout (cKO) mice

TAZ global KO mice were kindly provided by Prof. Ximei Wu (School of Medicine, Zhejiang University, China). The detailed procedures for the generation of the mice were described previously.^19^ Osteoclast-specific *TAZ* cKO mice were generated, raised, and bred at the Model Animal Research Centre of Nanjing University. An overview of the osteoclast-specific cKO strategy using the CRISPR/Cas9 gene editing system is shown in Supplementary Fig. 4a. Two single guide RNAs (sgRNAs) targeting introns 2–3 and introns 3–4 of the *TAZ* gene were constructed and transcribed in vitro (S1: AAGATTGATCGTGATGGAGT S2: ACACACGTGAGCCTGCCCGC). A targeting donor vector was designed to replace exon 3 of the *TAZ* gene. Cas9 mRNA, sgRNA, and donor vector were mixed and injected into C57BL/6J mouse zygotes. The zygotes were transferred into the oviduct of pseudopregnant ICR female mice at 0.5 d post-coitum (dpc). All fetuses were collected from pregnant ICR females 19–21 d after transplantation of sgRNA-injected zygotes, and genotyping was conducted using PCR and sequencing of tail DNA to screen for positive F0 mice with a loxP-exon3-loxP sequence in the targeted *TAZ* gene. Positive F0 mice were crossed with C57BL/6J mice to generate *TAZ* heterozygous mice with loxP-flanked exon 3 (*TAZ*^*fl/+*^). Heterozygous mice were crossed to generate homozygous floxed mice (*TAZ*^*f/f*^). RANK-Cre mice were generated and supplied by Yasuhiro Kobayashi (Institute for Oral Science, Matsumoto Dental University, 1780 hiro-Oka Gobara Shiojiri, Nagano 390-0781, Japan).^40^ RANK-Cre mice were mated with *TAZ*^*f/f*^ mice to generate mice heterozygous for loxP-flanked exon 3 and heterozygous for the Cre transgene (heterozygous cKO mice; *TAZ*^*f/+*^*RANK*^*Cre*^). *TAZ*^*f/+*^*RANK*^*Cre*^ mice were then mated back to homozygous *TAZ*^*f/f*^ mice to generate homozygous *TAZ*^*f/f*^*RANK*^*Cre*^ cKO mice. Homozygous *TAZ*^*f/f*^ mice with no Cre transgene were used as WT littermate controls.

### OVX-induced bone loss

For the OVX-induced bone loss model, twelve 8-week-old female C57BL/6 mice were randomly divided into two groups: sham-operated controls and bilateral ovariectomy (OVX). The mice were anesthetized and underwent bilateral ovariectomy or sham operation. After eight weeks, femora, tibias, foreleg bones and vertebral bones were harvested.

### Micro-CT analysis

3D reconstructions of tibias and calvarias were completed as previously described.^41^ The bone morphometric parameters analyzed included BV/TV, Tb.N, Tb.Th and Tb. For femurs, Sp. Ct. BV/TV, and Ct. Th were analyzed. The skull suture area was analyzed for calvarial bones.

### Bone serum markers

Serum markers of bone turnover, including TRAP, CTX-I, and P1NP, were measured using ELISA kits (Cusabio, Houston, TX, USA) according to the manufacturer’s instructions.

### Bone histology and histomorphometry

For dynamic histomorphometric analysis, calcein green (15 mg·kg^−1^ body weight) was injected intraperitoneally into 6-week-old *TAZ*^*+/+*^, *TAZ*^*−/−*^, *TAZ*^*f/f*^, *and TAZ*^*f/f*^*RANK*^*Cre*^ cKO mice. Alizarin red (15 mg·kg^−1^ body weight) was injected 7 d later. The mice were sacrificed 1 week later at 8 weeks of age. After fixation in 10% formaldehyde for 3 d, undecalcified femurs were dehydrated in increasing concentrations of ethanol, cleared in xylene and embedded in methyl methacrylate (MMA). MAR was analyzed for each sample using BIOQUANT OSTEO Image Analysis Software (BIOQUANT, Nashville, TN, USA).

Static histomorphometric parameters of bone volume over total bone (BV/TV), osteoclast number (OC.N/BS), and osteoclast surface per bone surface (OC.S/BS) were acquired from PFA-fixed, paraffin-embedded, decalcified bone sections stained with H&E or TRAP. Histological images were captured using an Eclipse TS100 light microscope (Nikon Corporation, Japan) and analyzed using ImageJ software (NIH, Bethesda, MD, USA).

### Mechanical testing

Three-point bending tests were performed on the mid-shaft of the right fresh tibial bones obtained from all mice on a model 3366 Dynamic Mechanical Analyser (Instron, Norwood, MA, USA) as previously described.^41^

### Primary osteoclast culture, cell proliferation assays, and bone resorption assays

Primary osteoclast culture and bone resorption assays were performed as described in our previous study.^42^ All experiments were performed in triplicate.

### Inhibitor, plasmids, lentivirus and antibodies

The TAK1 inhibitor takinib (#1111556-37-6) was purchased from Sigma-Aldrich. TAZ overexpression lentiviral particles (TAZ-Flag: pLenti-EF1A-WWTR1-CMV-Flag-Puro) and TAK1 overexpression lentiviral particles (TAK1-Flag: pLenti-EF1A-TAK1-CMV-Flag-Puro) were generated by PPL (GeneBio Technology Inc. Nanjing, China). TAZ silencing lentivirus (shTAZ: pPLK/GFP^+^Puro-TAZ) was purchased from Shanghai Genechem Co. Ltd. (Shanghai, China). Silencing of shTAZ was performed with the following sequences: (1) 5ʹ-CCGGGCGATGAATCAGCCTCTGAATCTCGAGATTCAGAGGCTGATTCATCGCTTTTTG-3ʹ; (2) 5ʹ-CCGGGCGTTCTTGTGACAGATTATACTCGAGTATAATCTGTCACAAGAACGCTTTTTG-3ʹ; and (3) 5ʹ-CCGGCCAGGAACAAACGTTGACTTACTCGAGTAAGTCAACGTTTGTTCCTGGTTTTTG-3ʹ. Specific antibodies against β-actin (AC-15) (sc-69879) were purchased from Santa Cruz Biotechnology (Dallas, TX, USA). Antibodies against p-TAZ (#59971), TAK1 (#D94D7), p-TAK1 (#9339), I-κBα (#5209), p65 NF-κB (#8242) and p-p65 NF-κB (#3033) were purchased from Cell Signaling Technology (Danvers, MA, USA). Antibodies against TAZ (23306-1-AP) and Flag (66008-2-Ig) were purchased from Proteintech (Rosemont, IL, USA), and NFATc1 (#6677), antibodies against p-p65 NF-κB (#5088) were purchased from Bioworld Technology Inc. (St. Louis Park, MN, USA), and antibodies against TAZ (#242313) was purchased from Abcam (Cambridge, UK).

### Gene expression analysis and immunoblot analysis

Quantitative real-time PCR was performed with synthesized mouse cDNA as the amplification template and specific primers (human and mouse primer sequences are presented in Supplementary Tables 4 and 5, respectively). Real-time PCR and immunoblot analysis were performed as previously described.^42^ Western blots always included three independent samples. The statistical data are provided in Supplementary Table 6.

### Primary osteoclast culture, cell proliferation assays, and bone resorption assays

Primary osteoclast culture and cell proliferation and bone resorption assays were completed in triplicate as previously described.^42^

### Immunofluorescence and immunohistochemical staining

Immunofluorescence staining for TAZ and TAK1 was performed as described in a previous study.^41^ The primary antibody against TAZ and TAK1 was diluted to 1:200. Immunohistochemical staining for p-TAK1, p-p65, and NFATc1 was performed as described in a previous study.^43^ The primary antibodies against p-TAK1, p-p65, and NFATc1 were diluted 1:100.

### Coimmunoprecipitation (co-IP)

For co-IP, total BMM proteins were extracted from transduced cells using NP40 lysis buffer, and the protein concentration was quantified. A total of 500 µg of protein lysate was precleared with either Protein A-agarose or Protein G-agarose beads (Sigma-Aldrich) and then incubated with anti-Flag M2 agarose beads (Sigma-Aldrich) overnight at 4 °C with gentle agitation. Flag-fusion proteins were eluted from the anti-Flag M2 agarose beads with SDS-PAGE sample loading buffer and boiled for 5 min. The samples were centrifuged at 13 000 r·min^−1^ for 5 min, and the supernatants were collected and subjected to SDS-PAGE and western blot analysis as described above.

### Statistics

Quantitative data are presented as the mean and standard deviation. Student’s *t* test for two groups and one-way or two-way ANOVA followed by Tukey’s test for multiple groups was applied to evaluate differences. Statistical significance levels are represented as *P < 0.05, **P < 0.01, and ***P < 0.001. SPSS Statistics for Windows (version 19.0, IBM Corp, Armonk, NY, USA) was used for all analyses.

## Supplementary information

Supplementary Figures and tables
