# Supplementary material for: TAZ inhibits osteoclastogenesis by attenuating TAK1/NF-κB signaling
Source: Bone Res. 2021 Jul 12;9:33. doi: 10.1038/s41413-021-00151-3 (PMC8275679; doi:10.1038/s41413-021-00151-3)
Supplement: Supplementary file 1 — Supplementary Figures and tables [file 41413_2021_151_MOESM1_ESM.pdf]

Supplementary Figures and figure legends

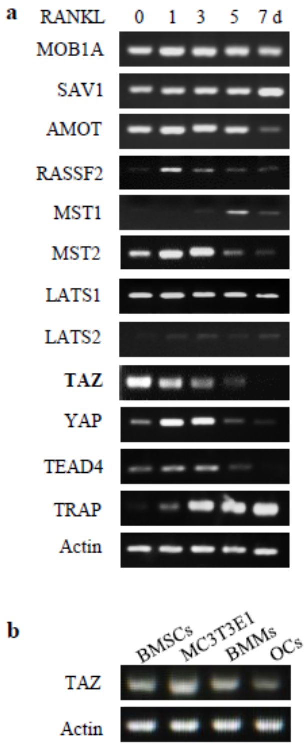

Supplementary Figure 1. (a) Semi-quantitative PCR gene expression profiling of key signaling molecules of the Hippo signaling pathway from BMM-derived osteoclasts stimulated with RANKL for 1, 3, 5, and 7 days. Unstimulated BMMs were used as basal mock control. **(b)** Semi-quantitative PCR gene expression profiling of TAZ from bone marrow stem cells (BMSCs), MC3T3E1 osteoblasts, BMMs and osteoclasts (OCs).

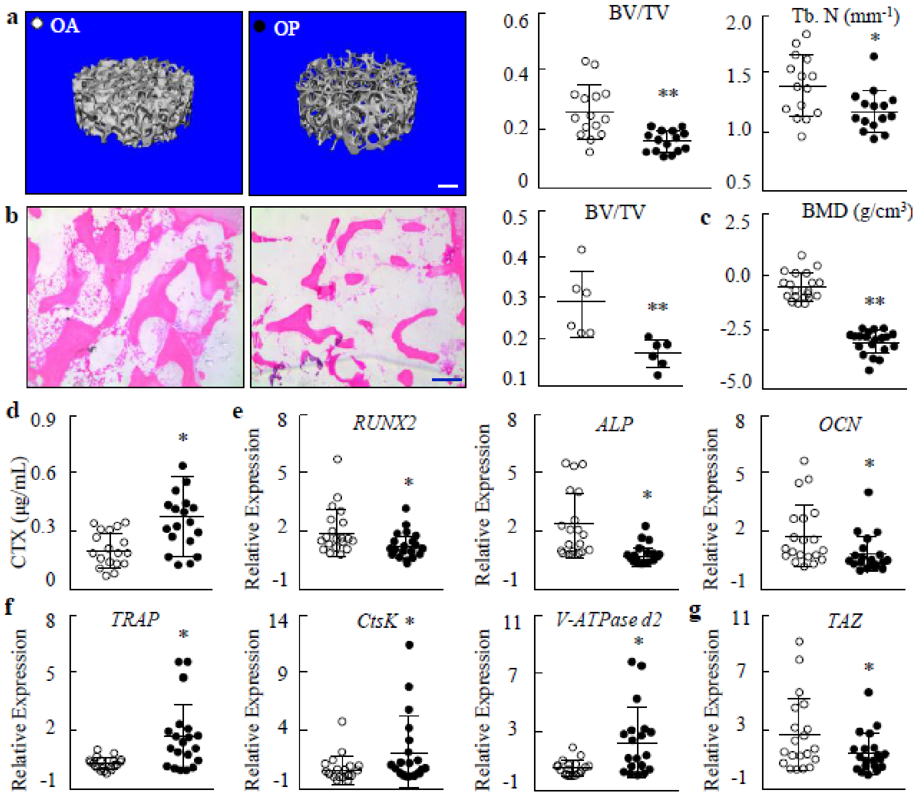

Supplementary Figure 2. Expression of TAZ is downregulated in human osteoporosis. **(a)** Three-dimensional micro-CT reconstructions and quantitative histomorphometric assessment of the femoral head from patients with osteoarthritis (OA, control) or osteoporosis (OP) ; n = 15. Scale bar denotes 1 mm. **(b)** H&E staining and quantitative histomorphometric assessment of femoral head tissue sections from OA and OP patients. Scale bar 1 μm. (n = 6). **(c)** BMD of the OA and OP femurs by dual-energy x-ray absorptiometry scan. **(d)** ELISA assessment of serum CTX levels in the OA and OP patients. **(e)** and **(f)** Real-time qPCR analysis of the expression of osteoblast marker genes **(e)** and osteoclast marker genes **(f)** from the OA and OP femoral heads, respectively. **(g)** Real-time qPCR analysis of the expression of the TAZ gene from the OA and OP femoral heads. E-G, n = 20. ○ and ● represents OA and OP group, respectively. Unpaired student's *t*-test was performed. *$p < 0.05$, and **$p < 0.01$.

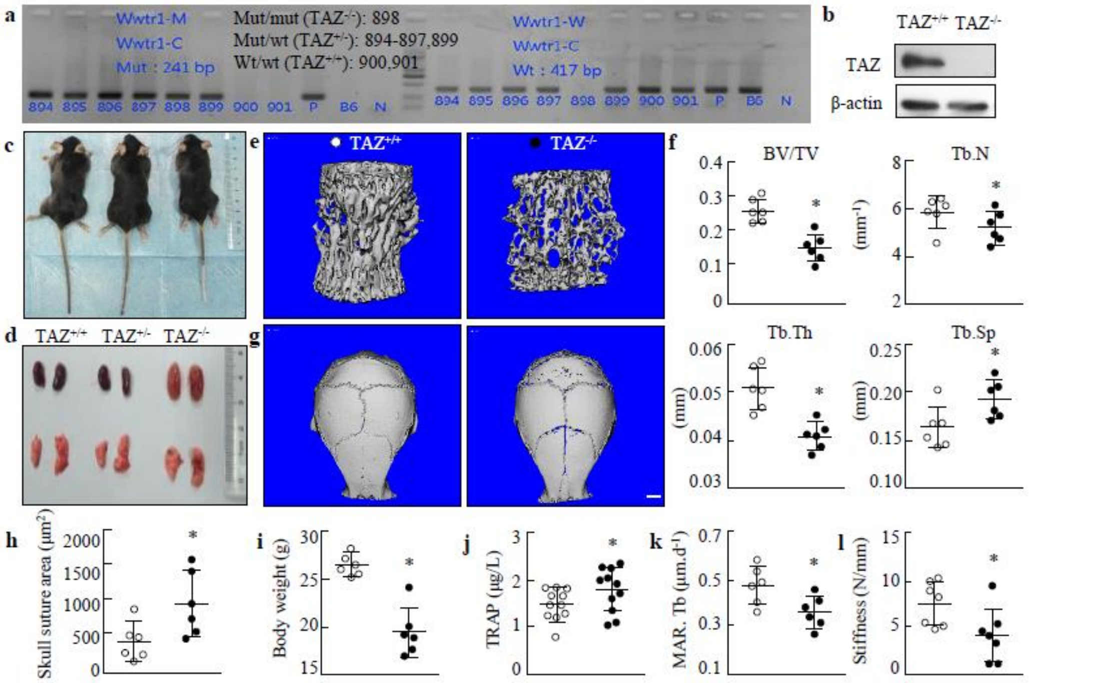

Supplementary Figure 3. TAZ global knockout exhibits osteoporotic phenotype. **(a)** Genotyping of *TAZ*$^{+/+}$ WT, *TAZ*$^{+/-}$ heterozygous, and *TAZ*$^{-/-}$ KO mice. **(b)** Efficiency of TAZ knockout in *TAZ*$^{-/-}$ BMMs. **(c)** Gross appearance of *TAZ*$^{+/+}$, *TAZ*$^{+/-}$ and *TAZ*$^{-/-}$ mice at 8 weeks of age. **(d)** Gross anatomical appearance of the kidneys and lungs from 8-week-old *TAZ*$^{+/+}$, *TAZ*$^{+/-}$ and *TAZ*$^{-/-}$ mice. **(e and g)** Representative 3D micro-CT reconstructions of (e) the second lumbar and (g) calvaria from 8-week-old *TAZ*$^{+/+}$ and *TAZ*$^{-/-}$ mice. Scale bar 100 μm and 1mm respectively. **(f)** Quantitative morphometric measurements of second lumbar trabecular BV/TV, Tb.N, Tb.Th and Tb.Sp. **(h)** Quantitative morphometric measurement of skull suture area. **(i)** Body weight of 8-week-old *TAZ*$^{+/+}$ and *TAZ*$^{-/-}$ mice. **(j)** ELISA assessment of serum TRAP levels in *TAZ*$^{+/+}$ and *TAZ*$^{-/-}$ mice. n = 6. **(k)** MAR of trabecular bone by calcein-alizarin red double labeling. **(l)** Mechanical three-point bending tests of stiffness bearing of *TAZ*$^{+/+}$ and *TAZ*$^{-/-}$ tibial bone. ○ and ● represents *TAZ*$^{+/+}$ and *TAZ*$^{-/-}$ group, respectively. Unpaired student's *t*-test was performed. *$p < 0.05$.

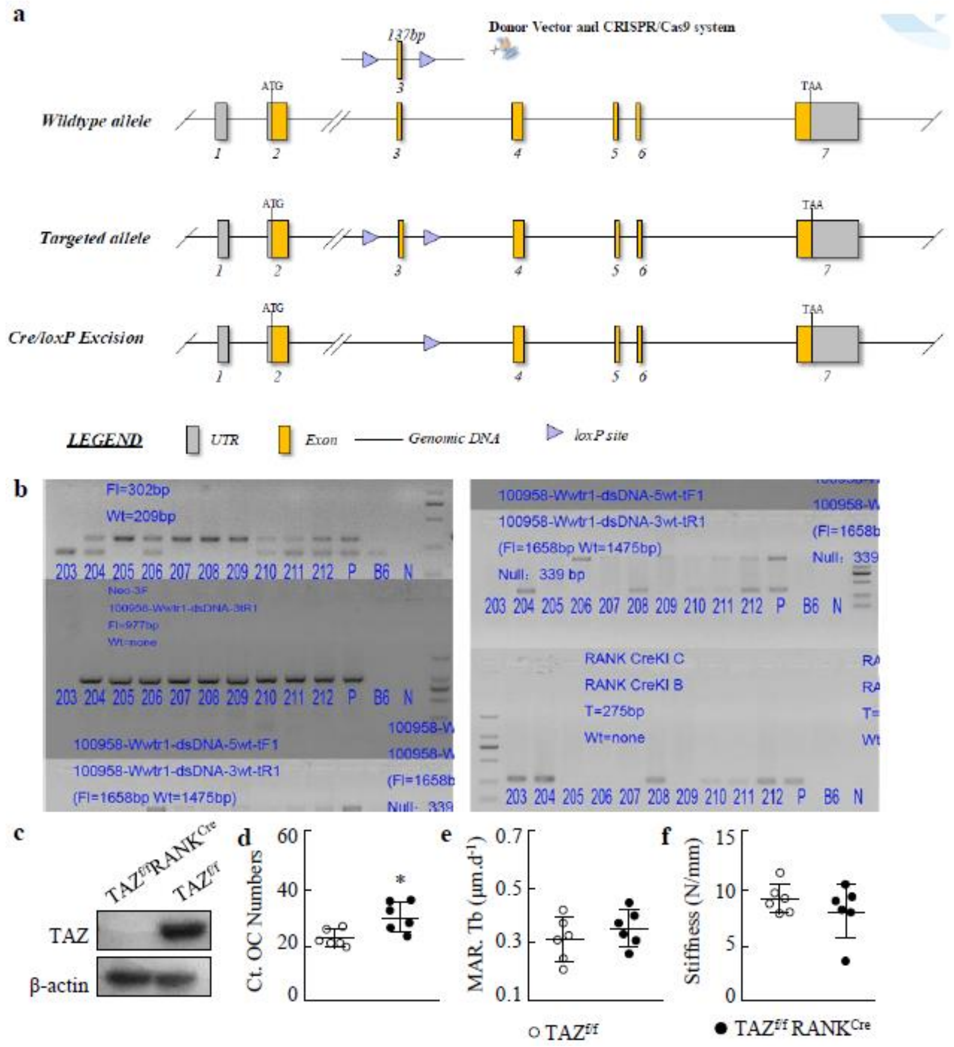

Supplementary Figure 4. Osteoclast-specific deletion of TAZ exhibits osteoporotic phenotype. **(a)** CRISPR/Cas9 strategy for the generation of TAZ$^{f/f}$ mice required for use subsequently to generate the conditional knockout of TAZ in osteoclast. **(b)** Genotyping of *TAZ*$^{f/f}$ and *TAZ*$^{f/f}$*RANK*^{Cre} mice, 205, 207 and 209 were genotyped as TAZ$^{f/f}$; 208 were genotyped as TAZ$^{f/f}$RANK^{Cre}. **(c)** Efficiency of TAZ knockout in *TAZ*$^{f/f}$*RANK*^{Cre} BMMs. **(d)** osteoclast numbers of cortical bone in *TAZ*$^{f/f}$ and *TAZ*$^{f/f}$*RANK*^{Cre}. **(e)** MAR of trabecular bone by calcein-alizarin red double labeling. **(f)** Mechanical three-point bending tests of stiffness bearing of *TAZ*$^{f/f}$ and *TAZ*$^{f/f}$*RANK*^{Cre} tibial bone. ○ and ● represents *TAZ*$^{f/f}$ and *TAZ*$^{f/f}$*RANK*^{Cre} group, respectively. Unpaired student's *t*-test was performed. *$p < 0.05$.

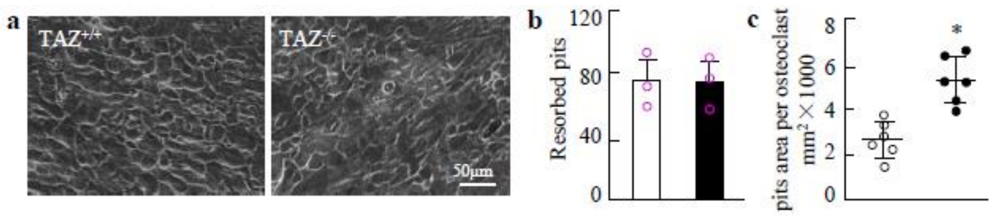

Supplementary Figure 5 Bone resorption for the equal numbers of mature TAZ$^{+/+}$ and TAZ$^{-/-}$ osteoclasts. **(a)**Scanning electron micrograph of bone resorption pits from osteoclasts derived from equal *TAZ*$^{+/+}$ and *TAZ*$^{-/-}$ osteoclasts stimulated with RANKL for 7 days. Scale bar 100 μm. **(b)** The numbers of bone resorption pits was quantified. **(c)** Average pit area of bone resorption. ○ and ● represents *TAZ*$^{f/f}$ and *TAZ*$^{f/f}$*RANK*^{Cre} group, respectively. Unpaired student's *t*-test was performed. $*p < 0.05$.

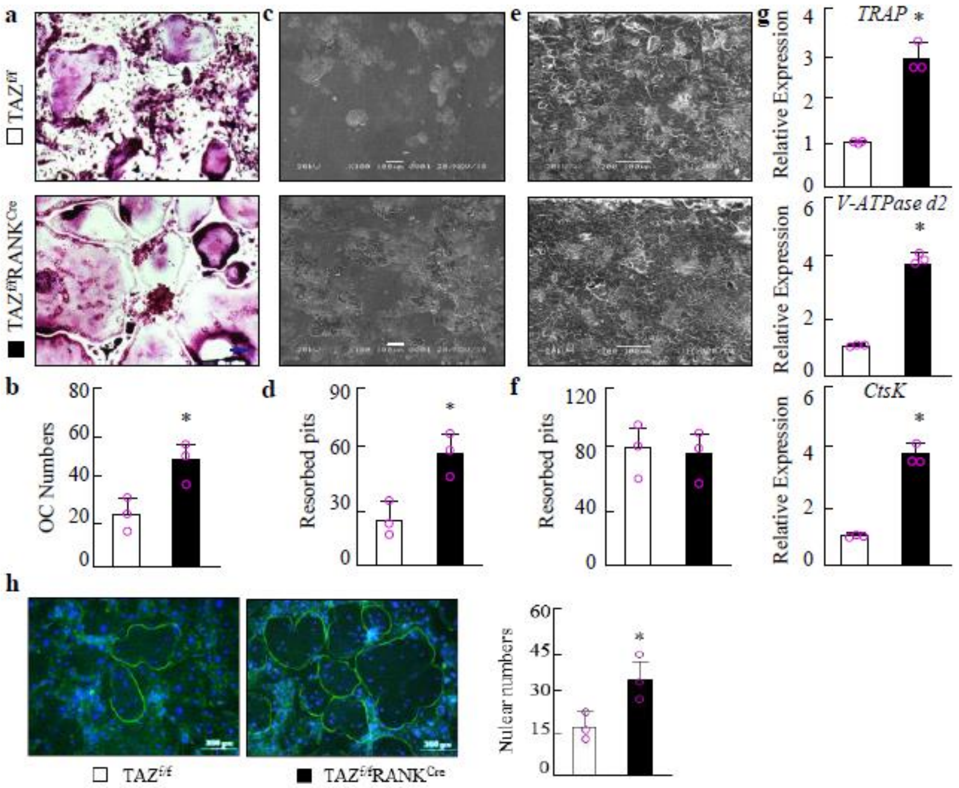

Supplementary Figure 6. Osteoclast-specific deletion of TAZ enhances RANKL-osteoclast formation and bone resorption. **(a)** TRAP stained images of osteoclasts derived from $TAZ^{f/f}$ and $TAZ^{f/f}RANK^{Cre}$ BMMs stimulated with RANKL for 7 days. Scale bar 10 μm. **(b)** The number of TRAP-positive multinucleated osteoclasts were quantified. **(c)** Scanning electron micrograph of bone resorption pits from osteoclasts derived from $TAZ^{f/f}$ and $TAZ^{f/f}RANK^{Cre}$ BMMs stimulated with RANKL for 8 days. Scale bar 100 μm. **(d)** The numbers of bone resorption pits was quantified. **(e)** Scanning electron micrograph of bone resorption pits from osteoclasts derived from equal $TAZ^{f/f}$ and $TAZ^{f/f}RANK^{Cre}$ osteoclasts stimulated with RANKL for 7 days. Scale bar 100 μm. **(f)** The numbers of bone resorption pits was quantified. **(g)** Real-time qPCR analysis of the expression of osteoclast marker genes *TRAP, CtsK*, and *V-ATPase d2* from $TAZ^{f/f}$ and $TAZ^{f/f}RANK^{Cre}$ BMMs stimulated with RANKL for 7 days. **(h)** F-actin/DAPI staining and statistic for the nuclear numbers per osteoclast $TAZ^{f/f}$ and $TAZ^{f/f}RANK^{Cre}$. □ and ■ represents $TAZ^{f/f}$ and $TAZ^{f/f}RANK^{Cre}$ group, respectively. n = 3 for each group. One-way ANOVA followed by a post-hoc Tukey test were performed. $^{**}p < 0.01$, and $^{***}p < 0.001$.

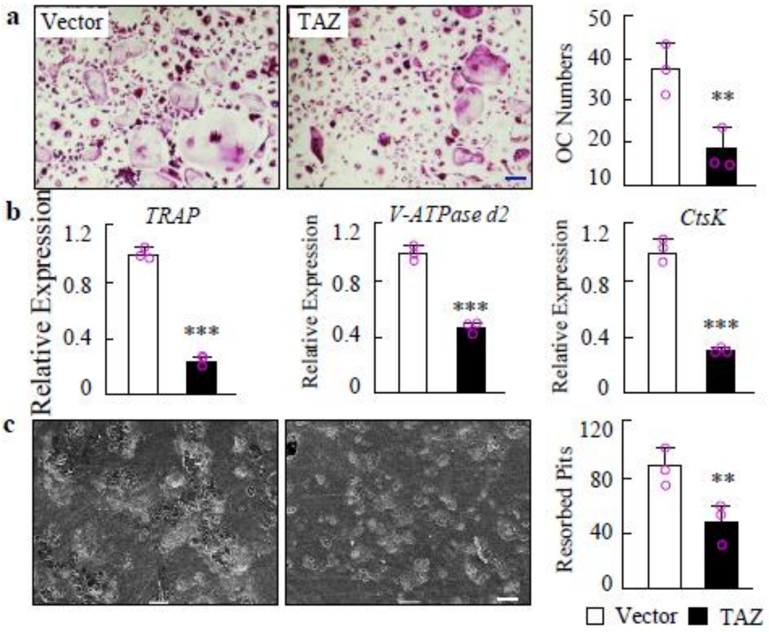

Supplementary Figure 7. Overexpression of TAZ suppressed RANKL-induced osteoclast differentiation and bone resorption. **(a)** TRAP stained and quantitative measurement of the number multinucleated osteoclasts derived from BMMs transduced with empty vector or TAZ overexpression lentiviral particles (TAZ) and then stimulated with 100 ng/mL RANKL for 7 days. Scale bar 10 μm. **(b)** Real-time qPCR analysis of the expression of osteoclast marker genes. **(c)** Scanning electron micrographs and quantitative measurement of the numbers of bone resorption pits. Scale bar 50 μm. n = 3 for each group. One-way ANOVA followed by a post-hoc Tukey test were performed. $^{**}p < 0.01$, and $^{***}p < 0.001$.

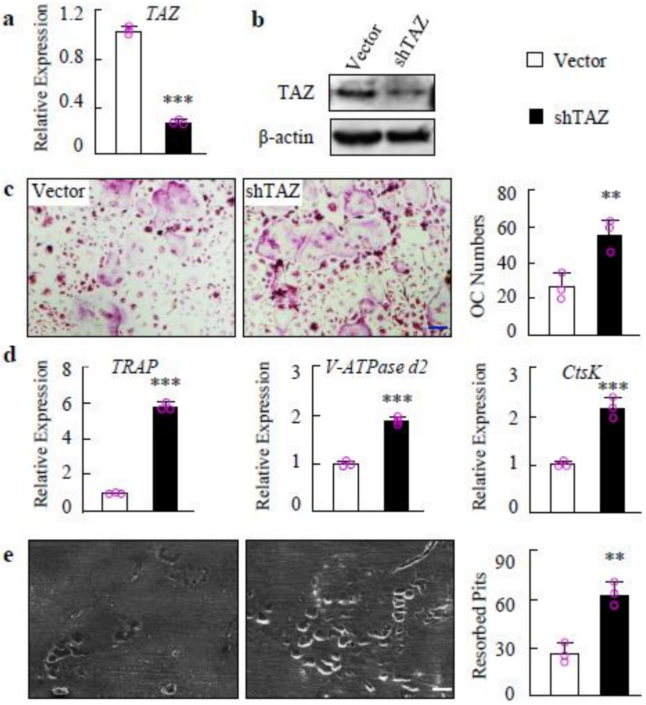

Supplementary Figure 8. Silence of TAZ enhanced RANKL-induced osteoclast differentiation and bone resorption. **(a)** silence efficiency of TAZ gene and **(b**) protein. **(c**) TRAP stained images and quantitative measurement of the number of multinucleated osteoclasts derived from BMMs transduced with control vector or TAZ silencing (shTAZ) lentiviral particles and then stimulated with RANKL for 7 days. Scale bar 10 μm. **(d)** Real-time qPCR analysis of the expression of osteoclast marker genes. **(e)** Scanning electron micrographs and quantitative measurement of the numbers of bone resorption pits. Scale bar 50 μm. One-way ANOVA followed by a post-hoc Tukey test were performed. $^{**}p < 0.01$, and $^{***}p < 0.001$.

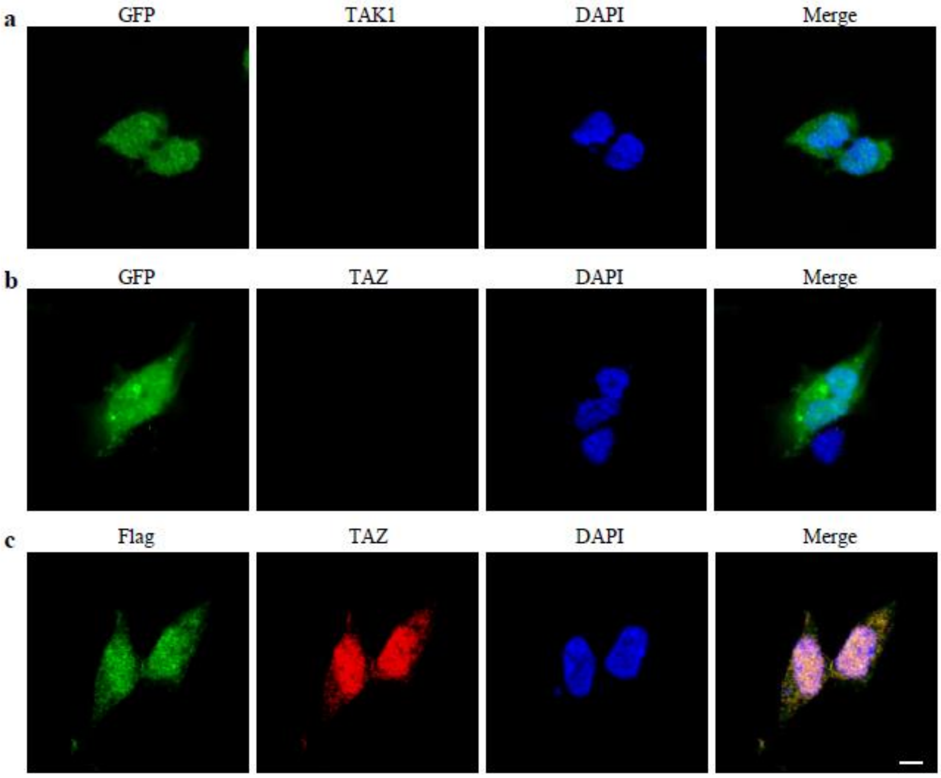

Supplementary Figure 9. TAK1 and TAZ antibody test**. (a)** Overexpression of TAK1-GFP in HEK293T and staining with TAK1 primary antibody and red second antibody. **(b)** Overexpression of TAZ-GFP in HEK293T and staining with TAZ primary antibody and red second antibody. **(c)** Overexpression of TAZ-Flag in HEK293T and staining with Flag and TAZ primary antibody and green and red second antibody, respectively. Scale bar = 10 μm.

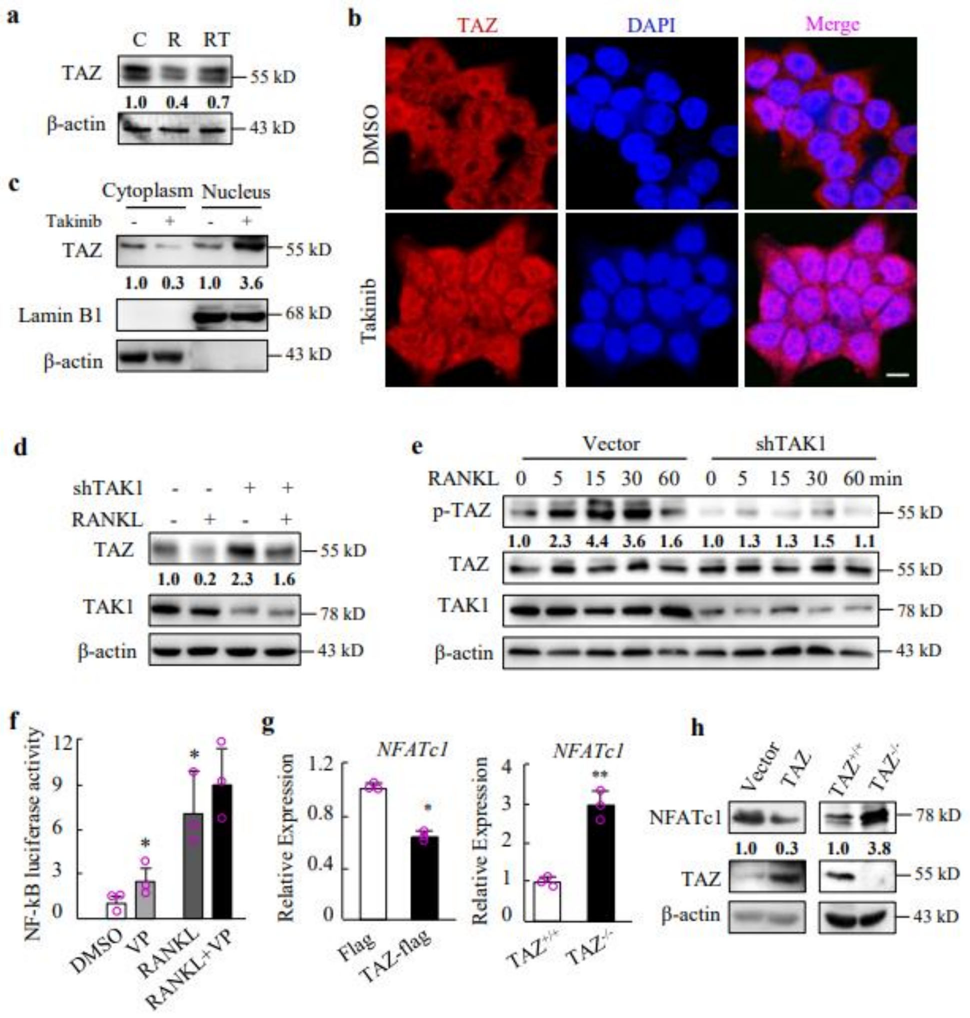

Supplementary Figure 10. Reciprocal suppression between TAZ and the NF-κB pathway regulates osteoclast differentiation. **(a)** Western blot analysis evaluating the effect of takinib on TAZ protein following RANKL stimulation for 48 h in BMMs. C, R, and RT represent control, RANKL, and RANKL + takinib, respectively. **(b)** TAZ immunofluorescence staining of HEK293T treated with DMSO or takinib. Scale bar = 10 μm. **(c)** Western blot analysis of TAZ localization to nuclear or cytoplasmic fractions in BMMs following stimulation with takinib. β-actin was used as marker for cytoplasmic fraction and Lamin B1 as marker for nuclear fraction. **(d)** Western blot analysis evaluating the effect of TAK1 silence on TAZ protein following RANKL stimulation for 48 h in BMMs. **(e)** Western blot analysis evaluating the effect of TAK1 silence on TAZ phosphorylation during RANKL stimulation in BMMs. **(f)** Luciferase analysis of NF-κB activity stimulated with verteporfin of TAZ inhibitor and RANKL in RAW264.7 for 6 h. **(g)** Real-time qPCR analysis of NFATc1 expression. **(h)** Western blot analysis of NFATc1 protein expression using total cellular proteins. One-way ANOVA was followed by post-hoc Tukey test. ** $p < 0.01$.

Supplementary Table 1. Patient Information

	Osteoarthritis (OA)	Osteoporosis (OP)
Number	20	20
Age (Year)	62.48±1.79	62.53±1.86
BMI	25.74±2.76	22.06±2.59*
**Basic Disease**		
Hypertension	7	4
Chronic Type-B Hepatitis	3	1
Fatty liver	3	1
Hyperuricemia	3	1
Other diseases	Cataract (1),<br>Cholecystolithiasis (1),<br>Asthma (1),<br>Hypohepatia (1)	Hepatic Cyst (3),<br>Gallbladder Polyps (2),

Unpaired student's *t*-test was performed. *$p < 0.05$.

Supplementary Table 2. Patient Information 2

	Normal bone density (N)	Osteoporosis (OP)
Number	6	6
Age (Year)	65.43±4.58	64.45±6.06
BMI	22.68±2.49	24.02±2.40*
BMD	-0.22±0.88	-3.38±0.71**

Unpaired student's *t*-test was performed. *$p < 0.05$, **$p < 0.01$.

Supplementary Table 3. TAZ global knockout breed number information

	Wildtype ($TAZ^{+/+}$)	Heterozygous ($TAZ^{+/-}$)	Knockout ($TAZ^{-/-}$)
Female	282	402	3
Male	198	286	18
Total	480	688	21
Embryo Genotype	17	31	16

Supplementary Table 4. Human Primer Pair Sequences

Genes	Forward (5’-3’)	Reverse (5’-3’)
TAZ	GGCTGGGAGATGACCTTCAC	CTGAGTGGGGTGGTTCTGCT
TRAP	GACTGTGCAGATCCTGGGTG	GGTCAGAGAATACGTCCTCAAAG
CtsK	TCCTTCCAGTTTTACAGCA	GTTTCCCCAGTTTTCTCC
V-ATPase d2	AAGGTGCGGAGCTGTACTTC	TCAGGTCTTCTAGGGTCTCACA
RUNX2	AGTTCCCAAGCATTTCATC	GGCAGGTAGGTGTGGTAGT
ALP	CCCTTCACTGCCATCCTGTA	GCCTGGTAGTTGTTGTGAGC
OCN	CACACTCCTCGCCCTATT	GGTCTCTTCACTACCTCGCT
β-actin	AGCCATGTACGTAGCCATCC	CTCTCAGCAGTGGTGGTGAA

Supplementary Table 5. Mice Primer Pair Sequences

Genes	Forward (5'-3')	Reverse (5'-3')
TAZ	GGCCCTATCATTCACGGGAG	TTGACGGTCATGGGTGTCTG
TRAP	TCCTGGCTCAAAAAGCAGTT	ACATAGCCCACACCGTTCTC
CtsK	CTTCCAATACGTGCAGCAGA	TCTTCAGGGCTTTCTCGTTC
V-ATPase [illegible]	AAGCCTTTGTTTGACGCTGT	TTCGATGCCTCTGTGAGATG
NFATc1	CAGCTGCCGTCGCACTCTGGTC	CCCGGCTGCCTTCCGTCTCAT
ALP	CTTCTTCTTGCTGGTGGA	AGGTGTCTTTCTGGGATGT
OCN	TTCAACGATCTGAGATTTGTGGG	GGATGAGGAATGCGCCCTA
MOB1A	GGGTTTATGCCCATATCTACCAC	CCAACTCACGCCTATCAATCAG
SAV1	ATGCTGTCCCGCAAGAAAAC	AATGAAGGCATGAGATTCCGC
AMOT	CCGCCAGAATACCCTTTCAAG	CTCATCAGTTGCCCCTCTGT
RASSF2	CCCTAATCCCATGCGGACAAG	GGAGTTGCAGGTTTTGACCTTC
MST1	AGCCCTCACGTAGTCAAGTAT	TCTTGTTCCGTAGCCGAATGATA
MST2	GCAAAACGCAACACTGTAATAGG	AGCCCTCATCGGATGTATATCAG
LATS1	AATTTGGCACACATCATAAAGCC	ACGAGGGTCTTGGTAACTCATT
LATS2	GGACCCCAGGAATGAGCAG	CCCTCGTAGTTTGCACCACC
YAP	TGAGATCCCTGATGATGTACCAC	TGTTGTTGTCTGATCGTTGTGAT
TEAD4	GAGCGACTCGGCAGATAAGC	CCACACGGCGGATAGATAGC
β-actin	AGCCATGTACGTAGCCATCC	CTCTCAGCAGTGGTGGTGAA
GAPDH	AATGGATTTGGACGCATTGGT	TTTGCACTGGTACGTGTTGAT

Supplementary Table 6. Quantitative statistical analysis for western blot

Figure	Mean ±SD											
F1e	1.00±0.00	0.86±0.03	0.83±0.05	0.84±0.04	0.79±0.05	0.67±0.04	0.67±0.03	0.56±0.04	0.54±0.02	0.44±0.03	0.19±0.01	0.25±0.03
F1f	1.00±0.00	0.96±0.03	0.83±0.05	0.98±0.06	0.89±0.06	0.64±0.01	0.71±0.03	0.44±0.01	0.51±0.02	0.42±0.00	0.28±0.01	0.22±0.02
F5c	1.00±0.00	1.20±0.09*	2.92±0.28*	2.41±0.22*	1.66±0.50							
F5d	1.00±0.00	0.54±0.08*	0.45±0.05*	0.24±0.13*	0.23±0.09*							
F5e	1.00±0.00	0.32±0.14*	0.22±0.06*									
F6a	1.00±0.00	2.01±0.37*	3.82±0.05*	2.19±0.37*	1.62±0.45	1.00±0.31	1.22±0.23	1.53±0.24	1.38±0.43	1.26±0.30		
F6b	1.00±0.00	0.52±0.14*	2.19±1.59	2.10±1.53	1.16±0.02	1.04±0.24	1.45±1.15	1.94±2.10				
F6d	p-TAK1	1.00±0.00	4.96±0.31*	1.59±0.81								
	TAZ	1.00±0.00	0.95±0.15	2.63±0.49*								
	I-κBα	1.00±0.00	0.23±0.10*	0.6±0.09*								
	p-p65	1.00±0.00	4.34±1.58*	1.07±0.16								
SF10b	cytoplasm	1.00±0.00	0.34±0.02*	nucleus	1.00±0.00	3.59±1.32*						
SF10c	1.00±0.00	0.42±0.01*	0.73±0.09*									
SF10d	1.00±0.00	0.24±0.05*	2.26±0.14	1.58±0.24*								
SF10e	1.00±0.00	2.34±0.52*	4.49±1.19*	3.59±1.04*	1.64±0.49	1.04±0.13	1.37±0.54					
SF10h	1.00±0.00	0.28±0.08*	1.00±0.00	3.80±0.37*								

One or two-way ANOVA followed by a post-hoc Tukey test were performed. *$p < 0.05$.
